# Comparison of the Effects of Fucoidans on the Cell Viability of Tumor and Non-Tumor Cell Lines

**DOI:** 10.3390/md17080441

**Published:** 2019-07-26

**Authors:** Kaya Saskia Bittkau, Philipp Dörschmann, Martina Blümel, Deniz Tasdemir, Johann Roider, Alexa Klettner, Susanne Alban

**Affiliations:** 1 Pharmaceutical Institute, Kiel University, Gutenbergstraße 76, 24118 Kiel, Germany; 2 Department of Ophthalmology, University of Kiel, University Medical Center, Arnold-Heller-Str. 3, Haus 25, 24105 Kiel, Germany; 3 GEOMAR Centre for Marine Biotechnology (GEOMAR-Biotech), Research Unit Marine Natural Products Chemistry, GEOMAR Helmholtz Centre for Ocean Research Kiel, Am Kiel-Kanal 44, 24106 Kiel, Germany; 4 Faculty of Mathematics and Natural Sciences, Kiel University, Christian-Albrechts-Platz 4, 24118 Kiel, Germany

**Keywords:** *Fucus vesiculosus*, *Fucus serratus*, *Fucus evanescens*, *Laminaria digitata*, *Saccharina latissima*, *Dictyosiphon foeniculaceus*, heparin, cancer, cytotoxic, antiproliferative

## Abstract

Fucoidans extracted from brown algae exert manifold biological activities paving the way for the development of numerous applications including treatments outside tumor therapy such as age-related macular degeneration or tissue engineering. In this study, we investigated the antiproliferative effects of fucoidans extracted from six different algae (*Fucus vesiculosus*, *F. serratus*, *F. distichus* subsp. *evanescens*, *Dictyosiphon foeniculaceus*, *Laminaria digitata*, *Saccharina latissima*) as well as three reference compounds (Sigma fucoidan, heparin, enoxaparin) on tumor (HL-60, Raji, HeLa, OMM-1, A-375, HCT-116, Hep G2) and non-tumor (ARPE-19, HaCaT) cell lines. All fucoidans were extracted according to a standardized procedure and tested in a commercially available MTS assay. Cell viability was measured after 24 h incubation with test compounds (1–100 µg/mL). Apart from few exceptions, fucoidans and heparins did not impair cell viability. In contrast, fucoidans significantly increased cell viability of suspension cell lines, but not of adherent cells. Fucoidans slightly increased viability of tumor cells and had no impact on the viability of non-tumor cells. The cell viability of HeLa and ARPE-19 cells negatively correlated with protein content and total phenolic content (TPC) of fucoidans, respectively. In summary, none of the tested fucoidans turned out to be anti-proliferative, rendering them interesting for future studies and applications.

## 1. Introduction

Cancer is one of the leading causes of death in industrial nations and a wide variety of types of cancer exists. Despite enhanced research interest and already existing medical treatments, the mortality rate is still very high, depending on the cancer type. A common complication in cancer patients and one of the leading causes of death is venous thromboembolism (VTE) [1]. The first choice in the prevention and treatment of cancer-associated thrombosis (CAT) is low molecular weight heparins (LMWH) [2,3]. Several clinical trials and meta-analyses have concluded that the use of LMWH may improve overall survival in cancer patients, particularly in those with early stage disease [4]. Although this clinical benefit still needs to be proven by sufficiently powered studies, a wealth of experimental data supports the antitumor and antimetastatic activities of heparins, whereby manifold mechanisms may be involved [5,6,7]. However, major obstacles to a potential use of heparins as anticancer drugs are their high bleeding risk and their animal origin, which is associated with resource limitations as well as contrary to the precautionary principle [8]. 

A potential alternative may be fucose-containing sulfated polysaccharides, the so-called fucoidans, extracted from brown algae. Fucoidans display an attractive array of bioactivities and potential applications including not only cancer inhibition, but also immune modulation, pathogen defense and numerous others (e.g., influence on elastase and collagenase with regard to inhibition of skin aging, treatment of inflammation as well as liver and kidney health) [9,10]. Many of their pharmacological effects may contribute to their antitumor and antimetastatic activities observed in animal experiments [11,12]. A substantial advantage of fucoidans over heparins is their relatively weak anticoagulant activity and thus low bleeding risk, whereas many of their other effects are stronger than those of heparins [13,14,15].

In general, one of the pivotal initial in vitro tests is the examination of the influence of potentially active agents such as fucoidans on cell viability. Whereas antiproliferative effects on tumor cell lines are favorable for antitumor therapies, cytotoxicity on non-cancerous cells is a big limitation for any lead compound, including fucoidans. Such toxicity may hamper other potential applications of fucoidans, for example their use in tissue engineering for improvement of bone vascularization during bone repair [16,17]. Another attractive field is cosmetics, as fucoidans have been described to stimulate the proliferation of dermal fibroblasts and inhibit enzymes that degrade the extracellular matrix [9]. Even in ophthalmology, fucoidans are of interest for their favorable effects for the treatment of age-related macular degeneration (AMD) [18,19]. 

Plenty of in vitro studies have been published describing antiproliferative or cytotoxic effects of fucoidans, especially on tumor cell lines. For example, Saravana et al. recently reported that a fucoidan from *Saccharina japonica* inhibited the proliferation of human myeloma and T-cell lymphoma cells as well as having cell growth inhibitory effects on SH-SY-5Y (neuroblastoma cells) and MKN-28 (adenocarcinoma cells) [20]. Jiang et al. showed the cytotoxic effect of a fucoidan from *Ascophyllum nodosum* on XC (rat sarcoma) and Vero cells (African green monkey kidney) using colony formation assay [21]. Moreover, fucoidans have been reported to induce apoptotic signaling pathways [11,22,23,24,25]. 

According to literature, the first commercially available fucoidan (*Fucus vesiculosus*) from Sigma-Aldrich is one of the most widely examined fucoidans. Interestingly, it has been shown to have divergent effects depending on the cell line used. For example, neither toxic nor antiproliferative effects were demonstrated when testing porcine or human retinal pigment epithelium (RPE) [18], whereas the cell viability of human colon cancer cells (HT29) decreased by incubation with fucoidan (*Fucus vesiculosus*) from Sigma-Aldrich [26]. 

Another reason for the conflicting published effects of fucoidans on cell viability is the high variability of experimental designs concerning e.g., incubation time, type of assay and applied concentrations. Especially the latter varies by several orders of magnitude [27,28]. Testing high concentrations (>100 µg/mL) may be relevant for special applications such as topical use or tissue engineering [29]. However, it has to be considered that biological effects of very high fucoidan concentrations may just represent laboratory artefacts, e.g., caused by high viscosity, binding and neutralizing of serum components or even limited solubility. 

Further, it has to be considered that the most frequently tested commercial fucoidan had pronounced batch variability in the past and its effects on cell viability correlated with its purity [14]. Finally, only a few studies compared the effects of fucoidans from different brown algal species [20,27,29,30] on cell viability so far, whereby the observed differences may sometimes result from different extraction and purification procedures, rather than the use of different algal material.

In the present study, we tested the cell viability effects of six fucoidans on nine different cell lines. The fucoidans were extracted from six different brown algal species by using the same extraction method. Cell viability effects were assessed using one standardized assay enabling direct comparison of the results. The extracted algal species were *Fucus vesiculosus*, *F. serratus*, *F. distichus* subsp. *evanescens*, *Dictyosiphon foeniculaceus*, *Laminaria digitata* and *Saccharina latissima* resulting in the fucoidans named FV, FS, FE, DF, LD and SL. Among the nine cell lines were two suspension cell lines (HL-60 (acute myeloid leukemia), Raji (Burkitt lymphoma)) and seven adherent cell lines [HeLa (cervix carcinoma), ARPE-19 (human RPE), OMM-1 (uveal melanoma), A-375 (skin melanoma), HCT-116 (colon carcinoma), Hep G2 (hepatocellular carcinoma), HaCaT (keratinocytes)], thus including a total of seven tumor cell lines (HL-60, Raji, HeLa, OMM-1, A-375, HCT-116, Hep G2) and two non-cancerous cell lines (ARPE-19 and HaCaT). After incubation for 24 h with the different fucoidans, the cell viability was measured using a commercial MTS assay according to a standardized protocol. As reference compounds three different commercially available substances, namely reference heparin as well as reference enoxaparin (a LMWH) and the commercial *Fucus vesiculosus* fucoidan, were additionally tested. The tests were performed to obtain comparative information on the suitability of the different fucoidans for further investigations. 

## 2. Results

**Figure 1:**
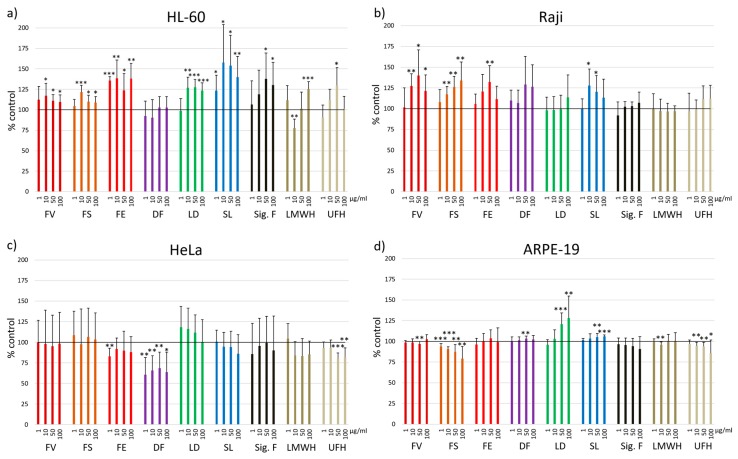

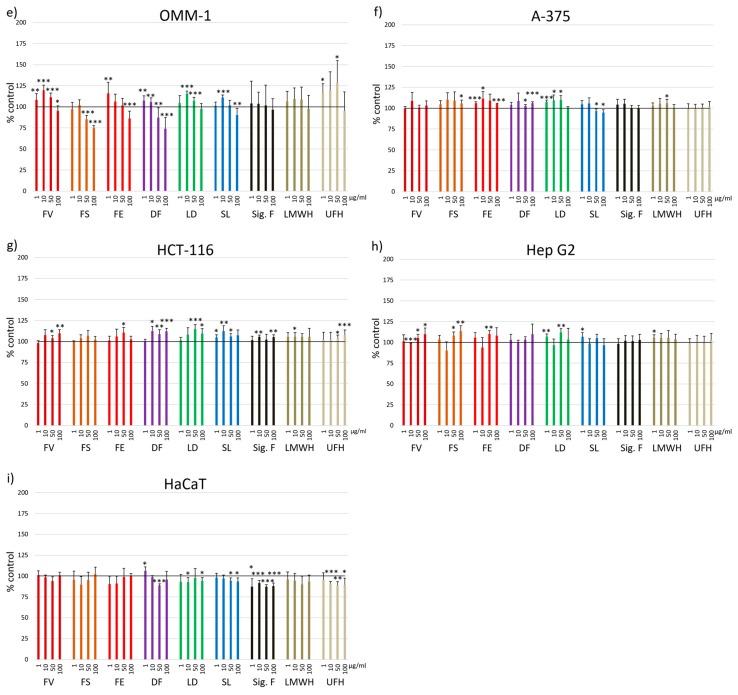
Cell viability was determined using MTS assay after 24 h incubation with 1, 10, 50 and 100 µg/mL fucoidan from *Fucus vesiculosus* (FV), *Fucus serratus* (FS), *Fucus distichus* subsp. *evanescens* (FE), *Dictyosiphon foeniculaceus* (DF), *Laminaria digitata* (LD) and *Saccharina latissima* (SL) as well as the reference substances Sigma-Aldrich fucoidan (*F. vesiculosus*) (Sig. F), enoxaparin (LMWH) and heparin (UFH). Nine different cell lines were tested: **a**) HL-60, **b**) Raji, **c**) HeLa, **d**) ARPE-19, **e**) OMM-1, **f**) A-375, **g**) HCT-116, **h**) Hep G2 and **i**) HaCaT. Values are expressed as mean and standard deviation in relation to untreated cells (100%). Significances compared to control were determined using student’s t-test * *p* < 0.05, ** *p* < 0.01, *** *p* < 0.001, n ≥ 4 × 3.

The details of the extraction procedure of the six fucoidans as well as their basic characteristics namely degree of sulfation (DS), molecular mass, monosaccharide composition, their contents of polyphenols, laminarin and protein as well as their radical-scavenging capacity, three exemplary bioactivities and their fluorescence intensity (FI) increasing effect on the sulfated glycan sensor Polymer-H are described elsewhere (unpublished data until accepted). To compare the effects of the different fucoidans and reference substances on cell viability, we determined their effects at concentrations up to 100 µg/mL (i.e., 0.07–0.53 µM fucoidans and 2.00 µM Sigma fucoidan, 7.69 µM heparin and 28.57 µM enoxaparin, whereby the molar concentrations can, however, only be very roughly estimated due to the polydispersity of these polysaccharides) on nine different cell lines in relation to the untreated control (100%) (Figure 1). 

In the leukemic cell line HL-60, all test compounds apart from the DF fucoidan increased the cell viability (Figure 1a). The effects of FE and SL were significant at all concentrations. FV, FS and LD increased the cell viability significantly at concentrations >1 µg/mL. Sigma fucoidan had stimulating effects at concentrations of 50 and 100 µg/mL (*p* < 0.05), heparin only at 50 µg/mL (*p* < 0.05) and the LMWH enoxaparin only at 100 µg/mL (*p* < 0.001). A questionable exception was the significant lower cell viability (77.7 ± 11.0%, *p* < 0.01) after treatment with 10 µg/mL enoxaparin, whereas 100 µg/mL increased the cell viability significantly (*p* < 0.001). The commercial fucoidan had a stimulating effect at 50 and 100 µg/mL (*p* < 0.05). 

In the Burkitt lymphoma cell line Raji, no inhibitory but rather a stimulatory effect was observed (Figure 1b). FV and FS fucoidans significantly increased the cell viability at 10, 50 and 100 µg/mL and FE only at 50 µg/mL (*p* < 0.01). The other fucoidans and the reference substances showed no significant effect.

The cervical adenocarcinoma cell line HeLa was one of the four cell lines, in which the compounds partly reduced the cell viability (Figure 1c). This was most pronounced with the DF extract at all concentrations (1 µg/mL 60.8 ± 20.7%, *p* < 0.01; 10 µg/mL 65.7 ± 17.8%, *p* < 0.01; 50 µg/mL 68.7 ± 19.2%, *p* < 0.01; 100 µg/mL 63.6 ± 24.3%, *p* < 0.05). FE fucoidan decreased the cell viability at 1 µg/mL (*p* < 0.01). The reference substance heparin showed significant reduction in cell viability at concentrations of 50 and 100 µg/mL (*p* < 0.001; *p* < 0.01).

In the RPE cell line ARPE-19, both stimulation and reduction of the cell viability was found. FS fucoidan significantly decreased it in a concentration-dependent manner (93.9 ± 3.3%, *p* < 0.001; 90.9 ± 2.6%, *p* < 0.001; 87.4 ± 8.7%, *p* < 0.01; 79.3 ± 14.7%, *p* < 0.01) (Figure 1d). In contrast, LD fucoidan concentration-dependently increased the cell viability (50 µg/mL *p* < 0.001; 100 µg/mL *p* < 0.01). The cell viability of the ARPE-19 cells incubated with the other compounds was comparable to the untreated control, although some small changes turned out to be significant (Figure 1d).

Similar to ARPE-19 cells, the uveal melanoma cell line OMM-1 responded differently to the treatment with the compounds, whereby FE and DF led to concentration-dependent effects (Figure 1e). The cell viability was significantly decreased by FS and DF at 50 (85.3 ± 4.6%, *p* < 0.001, 87.1 ± 11.9%, *p* < 0.01) and 100 µg/mL (74.6 ± 3.1%, *p* < 0.001, 74.3 ± 13.2%, *p* < 0.001) as well as by FE and SL at 100 µg/mL (86.3 ± 8.8%, *p* < 0.001, 90.4 ± 7.9%, *p* < 0.01). Significant increase in the cell viability was observed for FV, LD and SL fucoidans at 10 µg/mL. A trend of increase was further seen with heparin at concentrations of 1–50 µg/mL. 

In the skin melanoma cell line A-375 (Figure 1f) a significant increase of cell viability was observed for fucoidans from FE (1 µg/mL, 105.8 ± 1.9%, *p* < 0.001, and 100 µg/mL, 105.2 ± 0.9%, *p* < 0.001) as well as DF at the highest concentration (100 µg/mL, 106.1 ± 1.7%, *p* < 0.001) and LD at the lowest concentration (1 µg/mL, 107.6 ± 2.0%, *p* < 0.001). Of note, slightly significant reduction of cell viability for A-375 was measured only for SL (50 µg/mL, 96.5 ± 2.6% and 100 µg/mL, 94.8 ± 3.3%, both *p* < 0.05). 

The colon carcinoma cell line HCT-116 treated with DF (10 µg/mL 112.5 ± 5.5%, *p* < 0.01; 50 µg/mL 108.7 ± 5.4%, *p* < 0.05; 100 µg/mL, 111.9 ± 3.8%, *p* < 0.001) and LD (50 µg/mL, 114.9 ± 5.2%, *p* < 0.001), as well as heparin at 100 µg/mL (*p* < 0.001) showed significantly increased cell viability. Lower but still slightly significant increasing effects on cell viability were observed for fucoidans from FV (100 µg/mL, *p* < 0.01), FE (50 µg/mL, *p* < 0.05), SL at the lowest three tested concentrations (1 µg/mL, *p* < 0.05; 10 µg/mL, *p* < 0.01 and 50 µg/mL, *p* < 0.05). A slight, but significant increase in cell viability was also measured for the Sigma fucoidan at 10 µg/mL and 100 µg/mL, *p* < 0.01; heparin 100 µg/mL, *p* < 0.05). 

The hepatocellular carcinoma cell line Hep G2 showed no significant change of viability after application of almost all reference compounds at all concentrations with one exception: enoxaparin at 1 µg/mL, *p* < 0.01. The same is true for DF fucoidan, for which no significant change of cell viability was observed at all test concentrations and also for SL, except an increase at the lowest test concentration (*p* < 0.05). A slightly significant increase in cell viability was however measured for fucoidans from all *Fucus* species, namely FV with increasing concentration (50 µg/mL and 100 µg/mL, both *p* < 0.05), FS at the two highest test concentrations (50 µg/mL, *p* < 0.05 and 100 µg/mL, *p* < 0.01), FE (50 µg/mL, *p* < 0.01) as well as LD (1 µg/mL and 50 µg/mL, both *p* < 0.01). 

In the non-cancerous keratinocyte cell line HaCaT, commercial fucoidan lowered the cell viability significantly at all used concentrations (1 µg/mL 87.1 ± 9.6%, *p* < 0.05; 10 µg/mL 91.8 ± 3.0%, *p* < 0.001; 50 µg/mL 87.2 ± 2.6%, *p* < 0.001; 100 µg/mL 87.9 ± 3.9%, *p* < 0.001). The heparins had differential effects: whereas no significant change in HaCaT viability was detected for LMWH, heparin showed a significant decrease at the three highest test concentrations (10 µg/mL, 91.2 ± 2.2%, *p* < 0.001, 50 µg/mL, 88.6 ± 4.8%, *p* < 0.01, 100 µg/mL, 89.7 ± 7.7%, *p* < 0.05). 

Slight, but significant reduction of cell viability was further found for fucoidan extracts from LD (10 µg/mL 92.8 ± 5.1%, 100 µg/mL, 94.2 ± 4.0%, both *p* < 0.05) and SL (50 µg/mL 94.3 ± 3.4%, 100 µg/mL, 93.3 ± 4.5%, both *p* < 0.05). Notably, DF fucoidan significantly reduced the proliferation at 50 µg/mL (88.9 ± 1.7%, *p* < 0.001).

**Figure 2:**
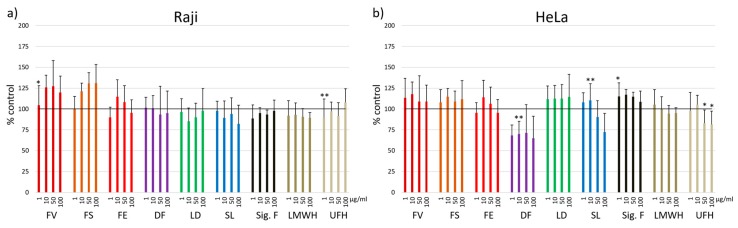
Cell viability was determined using MTS assay after 72 h incubation with 1, 10, 50 and 100 µg/mL fucoidan from *Fucus vesiculosus* (FV), *Fucus serratus* (FS), *Fucus distichus* subsp. *evanescens* (FE), *Dictyosiphon foeniculaceus* (DF), *Laminaria digitata* (LD) and *Saccharina latissima* (SL) as well as the reference substances Sigma-Aldrich fucoidan (*F. vesiculosus*) (Sig. F), enoxaparin (LMWH) and heparin (UFH). Two different cell lines were tested: **a**) Raji, **b**) HeLa. Values are expressed as mean and standard deviation in relation to untreated cells (100%). Significances compared to control were determined using student’s t-test * *p* < 0.05, ** *p* < 0.01, n = 3 × 3.

In addition to the incubation for 24 h, cell viability was estimated after 72 h for selected cell lines (Figure 2). In general, the cell viability is mostly comparable to control. In Raji cells, no significant changes were observed compared to control except for 1 µg/mL FV fucoidan and 1 µg/mL UFH (104.5 ± 1.9%, *p* < 0.05; 89.9 ± 2.1%, *p* < 0.01). In the HeLa cell line, the cell viability was significantly decreased with 10 µg/mL DF fucoidan (69.9 ± 10.2%, *p* < 0.01). UFH decreased viability compared to control at 50 and 100 µg/mL (82.8 ± 10.0% and 81.4 ± 8.6%, both *p* < 0.05). SL fucoidan significantly increased viability (10 µg/mL, 110.2 ± 2.8%, *p* < 0.01) as well as 1 µg/mL Sigma fucoidan (114.9 ± 8.7%, *p* < 0.05). The differences of Raji and HeLa cell viability between 24 h and 72 h incubation time compared to control are minor.

**Figure 3:**
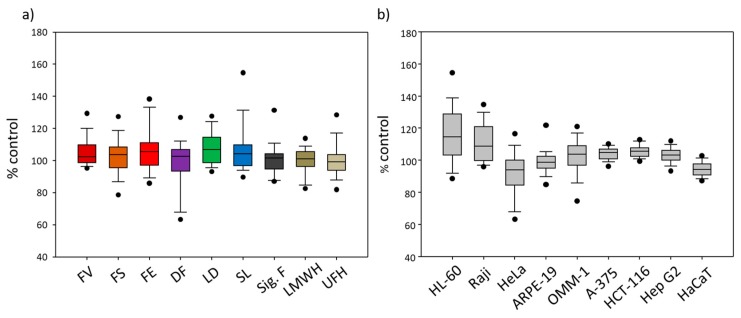
(**a**) Cell viability changes (all nine tested cell lines combined) in response to differently sourced fucoidans (i.e., from *Fucus vesiculosus* (FV), *Fucus serratus* (FS), *Fucus distichus* subsp. *evanescens* (FE), *Dictyosiphon foeniculaceus* (DF), *Laminaria digitata* (LD) and *Saccharina latissima* (SL)) as well as to the reference substances Sigma-Aldrich fucoidan (*F. vesiculosus*) (Sig. F), enoxaparin (LMWH) and heparin (UFH). (**b**) Cell viability changes (all nine test compounds combined, i.e., seven fucoidans, LMWH and UFH) depending on the cell line. The Whisker box plots are based on all the measured effects of each test compound (i.e., 1–100 µg/mL). The boxes represent the 25th percentile (boundary of the box closest to zero), the median (horizontal line in the box) and the 75th percentile (boundary of the box farthest from zero). Whiskers (error bars) above and below the box indicate the 95th and 5th percentiles. Outliers are shown as individual points.

Besides the comparison of individual effects of fucoidans on the nine cell lines, we aimed to evaluate whether the differently sourced fucoidans show different effects on cell viability as well as whether the cell lines differ in their response to all fucoidans and heparins (Figure 3). 

Regarding the medians (ranging from 99% to 107%), neither the fucoidans nor the heparins modified the cell viability (Figure 3a). The largest variability of cell viability was observed for SL (86–158%), followed by DF (61–129%), FS (75–134%) and FE (83–138%). DF and FS displayed the lowest cell viability at 61% and 75%, respectively, whereas SL, LD and FV stood out by their stimulating effects on cell viability (Figure 3a).

The effect of the fucoidans and heparins on cell viability differed stronger depending on the cell line than from each used fucoidan or heparin (Figure 3b). Concerning their medians, HeLa and HaCaT (94%) as well as HL-60 (115%) turned out to react more sensitively than the other cell lines. Overall, the test compounds did not lower the viability of any cell line, but displayed a slightly stimulating effect for the suspension cell lines HL-60 and Raji.

**Figure 4:**
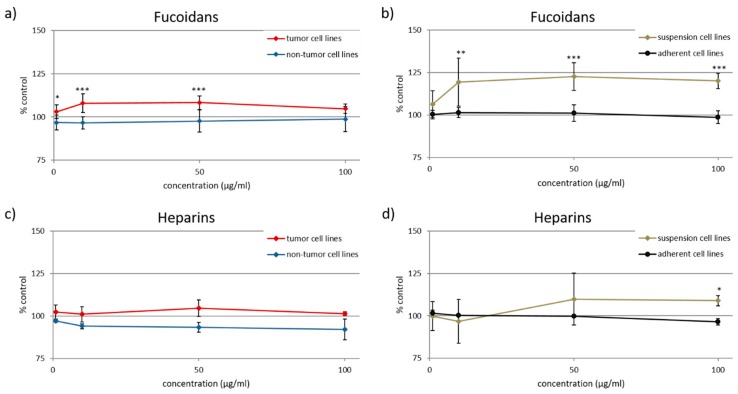
Cell viability effects of tested **a**), **b**) fucoidans and **c**), **d**) heparins in dependence on the type of cell line (Table 2). **a**) and **c**) show the mean cell viability of tumor and non-tumor cell lines and **b**) and **d**) that of suspension and adherent cell lines. Values are expressed as mean and standard deviation in relation to untreated control (100%). (n ≥ 4 × 3) Significances between tumor vs. non-tumor and suspension vs. adherent cell lines, respectively, were determined using student’s *t*-test * *p* < 0.05, ** *p* < 0.01, *** *p* < 0.001.

To compare the effects of fucoidans on tumor and non-tumor cell lines as well as on suspension cell lines and adherent cell lines (Table 2), the respective means of cell viability after treatment with all seven fucoidans including Sigma fucoidan were calculated. Whereas the fucoidans did not significantly modify the cell viability of the non-tumor cell lines (ARPE-19 and HaCaT), they slightly, but significantly increased that of the seven tumor cell lines (HL-60, Raji, HeLa, OMM-1, A-375, HCT-116 and Hep G2) (Figure 4a). At a concentration of 1 µg/mL, the tumor cell lines responded with 6% higher cell viability (*p* < 0.05), which further increased to 11 and 10% at 10 and 50 µg/mL (*p* < 0.001). Interestingly, the increasing effect of 6% at the highest concentration (100 µg/mL) was not significant (Figure 4a). Thus, the cell viability increase did not correlate with the concentration. 

The comparison of the suspension cell lines (HL-60 and Raji) with the adherent cell lines (HeLa, ARPE-19, OMM-1, A-375, HaCaT, HCT-116 and Hep G2) revealed a significant difference as well (Figure 4b). In contrast to any missing effect on the adherent cell lines, the fucoidans increased the cell viability of suspension cell lines to 119 ± 14% at the concentrations of 10 µg/mL (*p* < 0.01), to 123 ± 8% at 50 µg/mL (*p* < 0.001) and to 120 ± 5% at 100 µg/mL (*p* < 0.001). 

Both comparisons (Figure 4a,b) did not show any reduction of the viability of the cell lines incubated with fucoidans, leading to the conclusion that fucoidans are generally neither directly cytotoxic nor impair the cell viability. 

As controls, the corresponding comparisons were also performed with heparin and enoxaparin, which are known not to be cytotoxic [8] (Figure 4c,d). Although the respective differences were not significant, the results were similar to those obtained from the fucoidans. Significant was only the difference between the cell viability of the suspension and adherent cell lines incubated with 100 µg/mL heparins (suspension cell lines 108.9 ± 3.0%, adherent cell lines 96.4 ± 2.0%, *p* < 0.05) (Figure 4d). 

**Figure 5:**
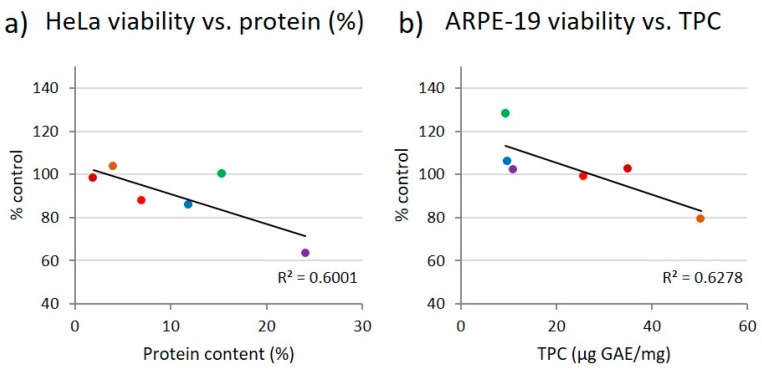
Correlations between viability of (**a**) HeLa cells and protein content of the six fucoidans and (**b**) ARPE-19 cells with total phenolic content (TPC). Colored dots indicate the corresponding values of test concentrations of 100 µg/mL *Fucus vesiculosus* (●), *Fucus serratus* (●), *Fucus distichus* subsp. *evanescens* (●), *Dictyosiphon foeniculaceus* (●), *Laminaria digitata* (●) and *Saccharina latissima* (●).

**Figure 6:**
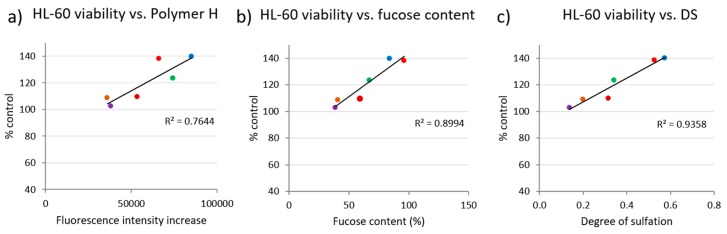
Correlations between viability of HL-60 cells and **a**) fluorescence intensity increase of Polymer-H, **b**) their fucose content and **c**) their degree of sulfation. Colored dots indicate the corresponding values of test concentrations of 100 µg/mL *Fucus vesiculosus* (●), *Fucus serratus* (●), *Fucus distichus* subsp. *evanescens* (●), *Dictyosiphon foeniculaceus* (●), *Laminaria digitata* (●) and *Saccharina latissima* (●).

In order to get indications of potential causes for the divergent reactions of the different cell lines in response to application of the different fucoidans, we performed correlation analyses between the cell viability at the highest fucoidan concentration (100 µg/mL) and the fucoidan characteristics protein content, total phenolic content (TPC) and fluorescence intensity (FI) increase of the glycan sulfate sensor Polymer H (unpublished data) for each cell line (Figure 5 and Figure 6). The fluorescence intensity of Polymer H (a synthetic polymer based on *ortho*-aminomethyl-phenylboronate units, ethylammonium units and dansyl units) is specifically increased in the presence of sulfated glycans. The so-called Polymer-H assay was shown to be an useful parameter for an initial quality screening of fucoidans [31,32] (unpublished data). Concerning the protein content, which was determined by means of the N-content, free proteins are not expected to be found due to the extraction and purification process. However, protein may be tightly associated with fucoidans as shown by previous studies [33,34].

For these correlation analyses, only those cell lines and fucoidan characteristics were selected wherein the effects of the six fucoidans on the cell viability differed by more than 25%. There were indeed some correlations between cell viability and fucoidan characteristics (Figure 5 and Figure 6). Negative correlations were observed between viability of HeLa cells and fucoidan protein content (R^2^ = 0.6001) (Figure 5a) as well as between viability of ARPE-19 cells and TPC (R^2^ = 0.6278) (Figure 5b). In contrast, the increase of the viability of HL-60 cells positively correlated with the FI increase of Polymer H (R^2^ = 0.7644) by the fucoidans. Additionally, HL-60 viability correlated with fucose content (R^2^ = 0.8994) and their DS (R^2^ = 0.9358) (Figure 6).

## 3. Discussion

Fucoidans extracted from brown algae have been described to exhibit antitumor effects [12,23,35], but also many other activities which could be beneficial for other potential applications like treatment of AMD, cosmetics and tissue regeneration [10,16,36]. Whereas antiproliferative effects on tumor cells are considered useful, reduction of cell viability or cytotoxic effects mostly constitute an obstacle to further development. Unfortunately, most studies investigated just one and sometimes poorly characterized fucoidan and results of various studies are not readily comparable due to different methods used for determining the parameters (e.g., cell line, assay type, concentrations). The most widely studied fucoidan is the *Fucus vesiculosus* fucoidan from Sigma Aldrich [11,14,15,23,26,36,37,38,39,40], which was demonstrated to vary in structural parameters (e.g., molecular weight) and purity (e.g., TPC) depending on the batch [14]. Consequently, data from studies that do not mention catalog number and lot number are hard to interpret. Fucoidans from different algal species have rarely been directly compared in cell viability studies. Another problem is the use of high fucoidan concentrations in cell experiments [27,37], which may considerably exceed those applied in vivo (e.g., therapeutic concentrations of heparins range from 5 to 15 µg/mL) and/or may lead to artificial effects.

Moreover, for the cell viability analyses reported in literature, many different assays were used such as WST-1, MTT, Alamar Blue, Annexin V/PI staining, cell cycle analysis, neutral red release assay, trypan blue exclusion assay, which not always deliver similar results [20,21,22,24,27,28,36,37,41]. To investigate whether different fucoidans vary in their effects on cell viability as well as whether the effects differ in dependency on the cell line, we tested the cell viability using the commercially available MTS assay in a standardized assay and analysis procedure. Adequate repetitions of experiments using different cell passages are essential to interpret the data plausibly. 

To come to a general statement about the influence of all tested fucoidans and the reference heparins on cell viability, we calculated the overall mean of all cell viability values after incubation with fucoidans (n = 7 × 9 × 4) and heparins (n = 2 × 9 × 4), respectively. The resulting means of 104.1 ± 13.1% and 100.5 ± 10.1% did not significantly differ from the control (data not shown). Additionally, in previous studies we did not find any cytotoxic effect of fucoidans measured by the release of lactate dehydrogenase (LDH) (data not shown). This leads to the conclusion that fucoidans just as heparins are non-toxic. To obtain an indication about longer incubation times, selected cell lines were treated with fucoidans and heparins for 72 h. Here, the effects were very comparable to those after 24 h of incubation, i.e., similar minor stimulating or decreasing effects on cell viability, respectively. Hence, our data show no indication of long term toxicity. However, as demonstrated by the results of this study, certain fucoidans and certain cell lines, respectively, may deviate from this general conclusion.

### 3.1. Cell Line Dependency

The results of the present study are compared with published studies investigating the effects of fucoidans on the same cell lines. As cancer cell lines are genetically unstable and often differ between laboratories, results obtained with these cell lines can be heterogeneous [42]. Therefore, caution has to be taken when comparing results of such experiments.

In the leukemia cell line HL-60 treated with fucoidans, no reduction but rather an increase of cell viability was detected (Figure 1a). In contrast, different studies described reduced cell viability of HL-60 cells treated with comparable concentrations of Sigma fucoidan (*F.v.*) for 48 h instead of 24 h, whereby they used neutral red release assay, MTT and WST-8 proliferation assay [38,39,43]. Whereas the Sigma fucoidan batch used as reference in this study turned out to be very pure (bought in 2018, indicated purity ≥ 95%), earlier studies used Sigma fucoidan of unknown purity [38], or crude extracts [39,43], which may explain the divergent results. Another reason could be the use of different assays, whereby the MTT and WST-8 proliferation assay are based on the same detection principle as MTS. Jin et al. and Atashrazm et al. additionally found a reduced cell viability of the leukemia cell line NB4, but not of K562 treated with Sigma fucoidan for 48 h [39,43], whereas Park et al. observed a reduced viability also in K562 cells [38]. After 24 h Jin et al. did not see a significant reduction in cell viability [39], which is comparable to our results. Accordingly, one cause for these different results could also be the different incubation times being associated e.g., with different availability of nutrients.

The influence of fucoidan purity on cell viability has previously been demonstrated by experiments with the Burkitt lymphoma cell line Raji: Crude Sigma fucoidan exerted an antiproliferative effect on Raji cells, whereas the fraction purified by ion exchange chromatography did not show such an effect [14]. Similarly, crude Sigma fucoidan had an antiproliferative effect, but fractions treated with H_2_O_2_ did not [15]. Moreover, Schneider et al. additionally tested fucoidan from SL, which was extracted with the same method as used in the current study [14]. Like the SL tested in the present study, SL fucoidan from the previous study contained considerably less coextracted phenolic compounds than the fucoidans from *Fucus* sp. and did not influence the proliferation [14]. 

Saravana et al. and Jiang et al. tested fucoidan from *Saccharina japonica* and *Ascophyllum nodosum* on the HeLa cell line which showed no significant effect on cell viability [20,21], whereas Sigma fucoidan (purity ≥ 95%) and abalone glycosidase-digested fucoidan from *Cladosiphon novae-caledoniae* inhibited the cell proliferation [20,35]. The effects on cell viability were thus dependent on the algae species, which fits to our results with HeLa, where DF fucoidan but none of the other test compounds displayed an antiproliferative effect. Regarding the discrepant findings by Saravana et al. on Sigma fucoidan, it has to be considered that they tested 1 mg/mL, whereas we examined 10–1000 fold lower concentrations [20]. 

In line with our data on ARPE-19 cells, Dithmer et al. and Li et al. observed no toxic effect of 100 µg/mL Sigma fucoidan on ARPE-19 cells [18,44]. Proliferative effects on other epithelial cells like endothelial progenitor cells (EPC) and human umbilical vein endothelial cells (HUVEC) were described for 100 µg/mL low-molecular-weight fucoidan from *Ascophyllum nodosum* [45]. In EPC a concentration-dependent increase of cell viability was detected [45] which fits with the effects of LD fucoidan in our study.

In the OMM-1 cell line, neither pure Sigma fucoidan (Figure 1) nor crude Sigma fucoidan had any effect on proliferation [36], whereas OMM2.5 and Mel270, two other uveal melanoma cell lines, reacted by activating and inhibiting proliferation, respectively [36]. It should additionally be mentioned, that OMM-1 was one of the cell lines, where we observed both proliferation stimulating (FV, FE, LD, SL) and inhibiting effects depending on the algal species and the concentration of the various fucoidans (FV, FS, FE, DF, SL). 

To our knowledge, the present study is the first one testing fucoidans on cell viability of A-375 skin melanoma cells, but there are some studies using B16 and RPMI-7951 skin melanoma cells. Similar to our results, Wang et al. found no effect on the cell viability of B16 cells treated with 100 µg/mL Sigma fucoidan (of undefined purity) for 48 h using the cell counting kit-8 (CCK8). This was confirmed by Ale et al. 2011, who tested crude Sigma fucoidan using MTT assay. However, 6–10 times higher concentrations reduced the B16 viability in both studies [46,47]. Vishchuk et al. tested fractions of 200 µg/mL fucoidans from *S. cichorioides*, *F. evanescens* and *U. pinnatifida* on the skin melanoma cell line RPMI-7951 and showed a slight reduction of cell viability after 24 h using MTS assay, which could be due to the higher concentration [30]. Furthermore, the cell viability of RPMI-7951 cells after 72 h was reduced, which could be explained by the extended incubation time [30].

A recent study of Park et al. reported the time- and concentration-dependent effects of crude Sigma fucoidan (Source: *F.v.*) on HCT-116 cells [48]. The concentration of 100 µg/mL fucoidan significantly reduced the viability by about 15–20% after 24 h incubation and about 50% after 48 h [48]. In the present study, neither the pure Sigma fucoidan nor any other of the test compounds reduced the viability of the HCT-116 cells, but on the contrary slightly increased cell viability.

Two studies on hepatocellular carcinoma cell line Hep G2 further underline the importance of concentration and purity of fucoidan regarding cellular effects [37,49]. In the study of Roshan et al. (2014), incubation with high concentrations (200–6000 µg/mL) of Sigma fucoidan for 24 h concentration-dependently reduced the cell proliferation and viability (by about 20% at 200 µg/mL) [37]. Zhurishkina et al. investigated both crude fucoidan from *F. vesiculosus* and two purified fractions obtained by ion-exchange chromatography [49]. In accordance with our results, treatment of Hep G2 cells with 120 µg/mL of the compounds for 24 h did not modify the cell viability and proliferation [49]. The same was found for the two purified fractions after incubation for 48 and 72 h, whereas the crude fucoidan reduced the cell viability [49].

Regarding HaCaT keratinocytes, Ryu et al. reported that fucoidan (unknown source) did not affect the cell viability up to 50 µg/mL, whereas 100 µg/mL fucoidan reduced it by about 10%, which corresponds to our findings on Sigma fucoidan [50]. A purified fraction from fucoidan extracted from *Turbinaria conoides* revealed, however, no cell viability reducing effect up to 1000 µg/mL [51].

Overall, the literature confirms that different cell lines react differently to fucoidans regarding cell viability (Figure 3b) and the influence of different fucoidans on cell viability varies as well (Figure 3a). Among the tested cell lines, HL-60 and Hela turned out as the most sensitive ones, whereby the viability of HL-60 was rather increased, whereas that of Hela was slightly reduced. 

### 3.2. Comparison of Non-Tumor and Tumor Cell Lines 

The viability of the two non-tumor cell lines ARPE-19 and HaCaT turned out to be not modified by the tested fucoidans (Figure 3b and Figure 4a). This is consistent with most of the literatureconcerning the effects of fucoidans on non-tumor cells [11,18,21,27,52,53,54,55]. No influence on the cell viability was reported for ARPE-19, WI-38 (human lung fibroblasts), Vero (African green monkey kidney), L929 (mouse fibroblasts), FHC (human normal colon epithelium), and RPE cells [11,18,52,54,55]. In human skin fibroblasts (HSF), a reduction in cell viability was only observed at a concentration of 5 mg/mL [53]. Gazha et al. reported that as much as 500 mg/mL fucoidan from *L. japonica*, *L. cichorioides* and *F. evanescens*, respectively, were required to induce apoptosis on human peripheral blood lymphocytes (PBL) [27]. 

In contrast to the non-tumor cell lines, the fucoidans slightly increased the viability of the seven tumor cell lines (Figure 3b and Figure 4a). However, this overall result was actually due to the stimulation of the proliferation of HL-60 and Raji cells. Most studies describe that the cell viability in tumor cell lines was decreased by fucoidans due to different mechanisms [11,20,23,24,28,37,41,51,54,55]. For example, antiapoptotic Bcl-2 was decreased by fucoidans [11,24,55]. Another example is cell cycle arrest such as G_0_ or SubG_1_ or G_1_ phase arrest [24,28,51]. Roshan et al. detected an increased generation of intracellular reactive oxygen species (ROS), which could be related with apoptosis induction [37]. The discrepant results between this study and the literature may be due to used cell line, fucoidan, purity of the fucoidan [11], concentration [20,23,24,37,41,51,55] and incubation time [11,20,23,28,51,54,55]. The relevance of the antiproliferative effects of fucoidans on tumor cells is currently unclear, since they exhibit numerous other effects contributing to their antitumor and antimetastatic activity (e.g., inhibition of angiogenesis, tumor cell migration, adhesion to extracellular matrix components and extracellular matrix degradation, antagonization of chemokines and adhesion molecules, stimulation of natural killer cells). 

### 3.3. Comparison of Suspension and Adherent Cell Lines

In total, we detected a stimulating effect of the fucoidans on the viability of the two suspension cell lines HL-60 and Raji, whereas the mean viability of the seven adherent cell lines was not modified (Figure 3b and Figure 4b). There are numerous studies investigating the influence of fucoidans on the viability of blood cancer cells including K562, NB4, THP-1, HS-Sultan, BCBL-1, TY-1, HL-60, U937 [23,25,38,39,40]. All of these studies reported either no or a reducing effect on the cell viability. In a previous study on Raji cells, fucoidan from *S. latissima* and purified Sigma fucoidan stimulated the cell proliferation measured using a BrdU-based proliferation ELISA, whereas the Sigma fucoidan (other batch than used in the present study) reduced the proliferation [13]. The stimulating effect was stronger after 48 h incubation than after 24 h and 72 h, however only significant for 150 µg/mL of the Sigma fucoidan after 48 h. Therefore, the question arises whether the increased cell viability measured with the MTS assay really results from increased proliferation or rather represents increased metabolic activity due to cell activation. This needs further investigations, whereby the best answer would be obtained by in vivo experiments on the antitumor activity of fucoidans in leukemia and lymphoma. It was previously described, that fucoidan fed mice developed lower tumor volume and tumor weight than the control mice after injection of blood cancer cells [43,56].

Regarding the adherent cells, the detection of any proliferation stimulating effect is impeded by the fact that further proliferation of confluent cells is restricted because of the limited growth area. 

### 3.4. Effect of Heparins on Cell Viability

According to the overall mean of the cell viability, the heparins (UFH, LMWH) showed no significant effects (100.5 ± 10.1%). Similar to the fucoidans, there were certain differences depending on the cell line, but even significant effects amounted to less than 22%. 

Heparin and enoxaparin, respectively, showed no effect on the proliferation on endothelial cells [57], human pulmonary epithelial cells A-549 and primary human osteoblasts [58,59]. This is comprehensible, as heparin is an endogenous compound of the human body. Further, heparins are well-known to exhibit antitumor and antimetastatic activity in vivo, but this is mainly due to other mechanisms than cytotoxic or antiproliferative effects [8]. Accordingly, heparin and enoxaparin turned out to have no influence on the viability of tumor cell lines [60,61]. However, in contrast to these findings, the viability of the human choriocarcinoma cell line JEG-3 was described to be stimulated [62], whereas the viability of three of four SCC (squamous cell carcinoma) cell lines after treatment with unfractionated heparin for 12, 24 and 48 h was reduced [63]. Such discrepant results underline that tumor cells may largely differ from each other and thus also in their reactions to any compounds. Heparan sulfate is described as a proliferation modulating substance and plays an important role in cancer diseases [64,65]. Saad et al. showed a decrease in cell viability of hepatocellular cell lines Hep G2 and HuH7 incubated with a mutant glypican 3 (heparan sulfate proteoglycan) without its GPI anchor [66]. On the other hand, it stimulated the growth of exponentially growing hepatocytes [67]. In general, heparan sulfate proteoglycans can act as positive or negative modulators of cell proliferation [67]. This depends on the cell type, the type of heparan sulfate and its interaction with for instance growth factors [66].

### 3.5. Correlation Analysis of Cell Viability and Characteristics of the Fucoidans

Analysis of the correlation between the cell viability and various characteristics of the six different fucoidans suggest that highly pure fucoidans do not impair the cell viability. The viability of HeLa cells negatively correlated with the protein content of the fucoidans (Figure 1c and Figure 5a) and that of ARPE-19 negatively correlated with the TPC (Figure 1d and Figure 5b). The latter is supported by the data for Sigma fucoidan having no antiproliferative effect (Figure 1g) as well as a low total phenolic content (data not shown, manuscript submitted). Some articles demonstrated that co-extracted compounds e.g., polyphenols are responsible for antiproliferative effects [14,15,68] and polyphenols extracted from brown algae were reported to exhibit pronounced antiproliferative effects [14,69]. Moreover, the viability of HL-60 cells positively correlated with the FI increase of Polymer-H, the DS and the fucose content of the fucoidans, whereby the viability was stimulated compared to the control. Polymer-H can be used as quality marker for fucoidans; its FI increase by fucoidans is related to their purity and biological activity ([32] manuscript submitted). The higher the fucose content and the DS of a crude fucoidan, the lower is its content of co-extracted compounds such as polyphenols, laminarin and alginic acid. Thus, a higher fucoidan purity was associated with an increase of viability of HL-60 cells. In general, well defined and highly purified fucoidans are an essential prerequisite for the development of medical applications.

Depending on the algae species the purity of the extracted crude fucoidans was shown to differ (manuscript submitted) and this characteristic turned out as the most important one for changes of the viability of the used cell lines. Due to the overall only small effects of the fucoidans on the cell viability, its impact was only observed in those three cell lines with the largest fucoidan-dependent variability. This may be also the reason for missing correlations between the cell viability and other parameters such as the molecular weight (data not shown), although that is generally considered an important parameter for the biological activities of fucoidans [15,70,71]. 

## 4. Materials and Methods 

### 4.1. Algal Material and Extraction Method

The algal material was kindly provided by the company Coastal Research & Management GmbH (Kiel, Germany). The used algae species for extraction, their harvest date and place are shown in Table 1. The fucoidans were extracted as previously described [34] and characterized (unpublished data). At first, Soxhlet extraction with 99% (*v*/*v*) ethanol was applied for defatting of the pulverized algal material. Then, the algal material was extracted using aqueous 2% calcium chloride for 2 h at 85 °C under reflux conditions. The supernatants of the raw extracts were concentrated by evaporation and precipitated with ice-cold ethanol in a final concentration of 60% at 4 °C. The precipitate was centrifuged, dissolved in demineralized water, dialyzed and lyophilized.

### 4.2. Reference Substances

As reference compounds fucoidan (*Fucus vesiculosus*) from Sigma Aldrich (Deisenhofen, Germany, purity ≥ 95%, Cat. No. F8190, Lot # SLBT5471), heparin (Cat. No. Y0001282) and enoxaparin (Cat. No. E0180000) from EDQM were purchased and solved in Ampuwa water (Fresenius Kabi, Schweinfurt, Germany).

### 4.3. Cell Culture

The human cell lines Raji (Burkitt lymphoma, ACC 319), HL-60 (acute promyelocytic leukemia, ACC 3), HCT-116 (colon carcinoma, ACC 581) and Hep G2 (hepatocellular carcinoma, ACC 180) were purchased from the German Collection of Microorganisms and Cell Cultures (DSMZ, Braunschweig, Germany). The HeLa cell line (cervical adenocarninoma) was a kind gift of Prof. Dr. Clement (Institute of Pharmacy, Kiel University, Germany). ARPE-19 cells (immortal human retinal epithelium) were bought from ATCC (CRL-2302TM, Manassas, VA, USA). The OMM-1 cell line (uveal melanoma, sub-cutaneous metastasis) was a kind gift of Dr. Sarah Coupland (North-West Cancer Research Institute, University of Liverpool, UK). The human malignant melanoma cell line A375 (skin melanoma) and the immortalized HaCaT cell line (keratinocytes) were purchased from Cell Line Service (Eppelheim, Germany).The characteristics of the used cell lines are shown in Table 2.

Raji, HL-60 and HeLa cell lines were cultivated in RPMI-1640 (Merck, Darmstadt, Germany, Cat. No. FG 1215, 11.11 mM D-Glucose) with 10% heat inactivated fetal bovine serum (FBS). The ARPE-19 cell line was cultivated in DMEM (Merck, Cat. No. FG0445, 25 mM D-glucose) with 10% FBS and 1% penicillin/streptomycin and 1% non-essential amino acids. OMM-1 cells were cultivated in RPMI-1640 (Merck, Cat. No. FG 1215, 11.11 mM D-glucose) with 10% FBS and 1% penicillin/streptomycin. HaCaT and Hep G2 cell lines were cultivated in Gibco RPMI-1640 (Thermo Fisher, Bremen, Germany, Cat. No 21875034, 11.11 mM D-glucose) medium supplemented with 10% FBS and 1% penicillin/streptomycin. The A-375 and HCT-116 cell line were cultivated in DMEM (Thermo Fisher, Cat. No. 11995065, 25 mM D-glucose) after adding 10% FBS and 1% penicillin/streptomycin. All cell lines were incubated in a humidified incubator at 37 °C and 5% CO_2_.

### 4.4. MTS Assay

The CellTiter 96^®^ AQueous One Solution Cell Proliferation Assay (Promega, Mannheim, Germany) was used to determine cell viability. The assay was performed in 96-well microplates in phenol red-free medium (HL-60, HeLa, Raji and OMM-1: RPMI 1640: Cat. No. F 1275, 11.11 mM D-glucose; ARPE-19: HyClone DMEM (GE Healthcare, Hamburg, Germany, Cat. No. SH30284.01, 25 mM D-glucose)). For HL-60, HeLa and Raji cells 1X Gibco GlutaMAX™-I (Life Technologies, Carlsbad, CA, USA, 35050-038) was added. The medium for ARPE-19 and OMM-1 was supplemented as described above in cell culture. For OMM-1 cells 2 mM L-glutamine (Merck, Cat. No. K0282) was added. For Hep G2, HaCaT, A-375 and HCT-116 cell lines the cell viability assay was also performed using Gibco RPMI 1640 medium as described above for cell culture. Extracts in medium were filtered through a 0.2 µm filter before treatment (Sarstedt, Nümbrecht, Germany, Cat. No. 83.1826.001). The Raji (10.000 cells/100 µL) and HL-60 (15.000 cells/100 µL) cells were seeded and treated with fucoidan extracts on the same day. ARPE-19 cells (10.000 cells/100 µL) were seeded and treated at 100% confluence. OMM-1 cells were treated at 80% confluence. HeLa (3000 cells/100 µL), Hep G2, HaCaT, A-375 and HCT-116 cells (each 10.000 cells/100µL) were seeded the day before treatment with fucoidan.

Cells were treated with the six different fucoidans as well as with the reference compounds (Sigma fucoidan, enoxaparin and heparin) in four different concentrations (1, 10, 50 and 100 µg/mL) for 24 h. As control, cells treated with 100 µL medium were used. After the incubation time 20 µL of the MTS solution were added to each well and incubated for 1 h at 37 °C, 5% CO_2_. The absorbance was measured at 490 nm. 

### 4.5. Statistical Analysis

Each experiment was performed in triplicates at least four times. Data of the experiments were analyzed statistically in Microsoft Excel using the mean, standard deviation and Student’s *t*-test. A Student’s *t*-test with *p* < 0.05 was considered as significant. All bars represent mean and standard deviation.

## 5. Conclusions

Due to the heterogeneous and partly conflicting data in the literature, the aim of the present study was to investigate the effects of different fucoidans on cell viability and to compare them depending on cell line and type (suspension/adherent, tumor/non-tumor cell lines). For this, six fucoidans extracted from different brown algal species (*Fucus vesiculosus*, *F. serratus*, *F. distichus* subsp. *evanescens*, *Dictyosiphon foeniculaceus*, *Laminaria digitata*, and *Saccharina latissima*) as well as three reference compounds (Sigma fucoidan, heparin, enoxaparin) were tested on nine different human cell lines (HL-60, Raji, HeLa, ARPE-19, OMM-1, A-375, HCT-116, Hep G2,and HaCaT). The cell viability was determined by the commercially available MTS assay according to a standardized test and analysis protocol for comparability of the results. After 24 h incubation of the cells with the fucoidans (1–100 µg/mL), the overall mean of the cell viability amounted to 104.1 ± 13.1% and was quite similar to that of the heparins (100.5 ± 10.1%), which are known to exhibit no antiproliferative effects. Although some individual cell viability values were significantly reduced compared to the control (at most of HeLa by DF, i.e., 39% reduction), no consistent antiproliferative effect was found for any fucoidan. The cell viability medians of the different fucoidans ranged from 99% to 107%, whereby the largest cell line- and concentration-dependent variability was observed for SL (86–158%) and DF (61%–129%). As indicated by the medians of the different cell lines ranging from 94% (HeLa and HaCaT) to 115% (HL-60), the cell lines differently reacted to fucoidans. Whereas cell viability of the adherent cell lines was not influenced by the fucoidans, that of the suspension cell lines (HL-60, Raji) was significantly increased. In contrast to the non-tumor cell lines (ARPE-19, HaCaT), the viability of the tumor cell lines was also slightly increased, but this was mainly due to the increased viability of HL-60 and Raji cells. Analyses on the role of the various analytical fucoidan characteristics for their marginally different cellular effects revealed that the effect on the cell viability is primarily dependent on the purity of the fucoidans including co-extracted compounds. The cell viability of HeLa and ARPE-19 cells negatively correlated with protein content and TPC, respectively, whereas that of HL-60 cells positively correlated with FI increase of Polymer H, fucose content and DS.

In summary, none of the tested fucoidans turned out to impair the cell viability, when tested at concentrations typical for systemic therapeutic applications. This makes them suitable for further investigations concerning many different applications. Despite the lack of antiproliferative effects on tumor cells, fucoidans still are of interest as potential antitumor agents due to numerous other antitumor mechanisms (e.g., inhibition of angiogenesis, tumor cell migration, tumor cell adhesion). 

## Figures and Tables

**Table 1:** Algal species, their harvest dates and sites.

Fucoidan	Algae Species	Harvest Date	Origin
SL	*Saccharina latissima*	July 2016	North Atlantic Ocean
DF	*Dictyosiphon foeniculaceus*	May 2017	Baltic Sea
LD	*Laminaria digitata*	June 2017	North Atlantic Ocean
FV	*Fucus vesiculosus*	July 2017	Baltic Sea
FS	*Fucus serratus*	July 2017	Baltic Sea
FE	*Fucus distichus* subsp. *evanescens*	July 2017	Baltic Sea

**Table 2:** Characteristics of the used cell line.

Cell Line	Cell Type	Suspension/Adherent	Tumor/Non-Tumor
HL-60	Acute promyelocytic leukemia	suspension	tumor
Raji	Burkitt lymphoma	suspension	tumor
HeLa	Cervical adenocarninoma	adherent	tumor
ARPE-19	Human retinal epithelium	adherent	non-tumor
OMM-1	Uveal melanoma	adherent	tumor
A-375	Skin melanoma	adherent	tumor
HCT-116	Colon carcinoma	adherent	tumor
Hep G2	Hepatocellular carcinoma	adherent	tumor
HaCaT	Keratinocytes	adherent	non-tumor
